# Editorial: Understanding in the human and the machine

**DOI:** 10.3389/fnsys.2022.1081112

**Published:** 2022-11-25

**Authors:** Yan M. Yufik, Karl J. Friston, Rosalyn J. Moran

**Affiliations:** ^1^Virtual Structures Research Inc., Potomac, MD, United States; ^2^Queen Square Institute of Neurology, University College London, London, United Kingdom; ^3^Department of Neuroimaging, Institute of Psychiatry, Psychology and Neuroscience, King's College London, London, United Kingdom

**Keywords:** self-organization, understanding, grasp, general intelligence, complexity, prediction, explanation, mental model

This Research Topic was initiated in a workshop—in August 2021 in Washington D.C. – under the auspices of the U.S. Air Force Office of Scientific Research and Air Force Research Laboratories. This Issue is dedicated to analyzing understanding and is a sequel to 2017 Research Topic, which focused on the fundaments of self-organization in the nervous system https://www.frontiersin.org/research-topics/4050/self-organization-in-the-nervous-system#articles. A crosscutting theme in both journals—and the workshop—is the principle of Variational Free-Energy Minimization (VFEM), also known as Active Inference (Friston et al., 2006; Friston, 2010; Parr et al., 2022). This principle has been applied to further our understanding of the role, adaptive value and neuronal mechanisms of the capacity to understand (“understanding of understanding”). Conceptualizing understanding as a product of uniquely human self-organization—obtaining levels of free energy minimization inaccessible to other species—appears to offer a promising perspective on the neuronal underpinnings of understanding and designing devices possessing a modicum of human understanding (machine understanding). This editorial reviews the state-of-affairs in the multidisciplinary domain of understanding R&D (“the science of understanding”), summarizes some key ideas in theoretical approaches centered on the application of VFEM (Yufik and Friston, 2016; Yufik et al., 2017), and introduces contributions in the present collection.

Human intellect apprehends the world and itself through the lens of understanding. Since the time of Aristotle (2004) the capacity to understand—and the innate desire to exercise that capacity—have been recognized as the defining features of human intelligence, distinguishing humans from other species (Lear, 1988; Greco, 2014). Analysis of how understanding operates and influences the ways humans interact with the world—and with each other—has remained a key focus in psychology (Piaget, 1974, 1978) and philosophical discourse throughout history [e.g., (Kant, 1990 (1781); Hegel, [1977 (1807)]; Locke, [1996 (1689)]; Russell, [1997 (1921)]; Descartes, [1998 (1637)]; Berkeley, [1998 (1734)]; Hume, [2018 (1739)]). Although never dormant, interest in the phenomenon of understanding was renewed and re-invigorated in the modern era, due to the emergence of radically novel conceptual constructs in mathematics, physics, biology, psychology and other disciplines turning to “eternal” questions like what makes the world understandable, the origins and limits of understanding, etc. from the realm of speculative philosophy to the mainstream of scientific inquiry (Mehra, 1999; Freeman, 2000; Barsalou, 2008; Rovelli, 2014; de Regt, 2017). Accomplishments in the last decades—at the intersection of computer science, neuroscience and other disciplines—have realized some intelligence (learning, reasoning) in engineering artifacts. The resulting proliferation of smart systems, including weapons capable of acting autonomously or collaboratively with warfighters, has created an urgent demand for advances in machine intelligence to furnish a competitive edge in commerce and defense. This Research Topic seeks to facilitate progress in the science of understanding, with a special focus on machine understanding.

What is understanding and how does it effect performance? Continuing debates on the subject (Gelepithis, 1986; Baumberger et al., 2016; Hannon, 2021) reveal a tangle of issues and controversies that can be traced back to Plato and Aristotle. And have not been settled since. In particular, difficulties persist in clarifying relations between understanding, knowledge and belief (Grimm, 2006; Baumberger, 2014; Pritchard, 2014), defining the value (benefits) of understanding in adaptive performance (Kvanvig, 2003, 2009; Grimm, 2012, 2014), circumscribing the relative roles of explanation and prediction enabled (and perhaps entailed) by understanding (Khalifa, 2013). The cognitivist school in psychology reduces understanding to possessing algorithms (subject S understands task T if S possesses algorithms for carrying out T) (Newell and Simon, 1972; Simon, 1979). Conversely, other authoritative sources maintain that understanding involves non-algorithmic and non-computable components (Penrose, 1997, 2016) and argue that algorithms can be designed so that computers give the impression of understanding a task, while remaining clueless about its meaning (Searle, 1990; Kauffman, 2010). An example from a psychology classic (Piaget, 1978) illustrates the distinction between the way non-algorithmic and algorithmic processes manifest: consider a row of N domino pieces standing on edge and compare two kinds of performance: predicting at a glance that, whatever N, when pushing the first piece, the last piece will fall, vs. predicting the same but only after having worked mentally through all the N pieces, one-at-a-time. According to our proposal, diverging views on understanding are not mutually exclusive but reflect different components and operational stages in the underlying mechanism, as discussed below.

Variational Free Energy Minimization (VFEM) rests on several assumptions, including the following: (a) to survive, any organism, from the simplest (bacteria) to most advanced (humans), must possess internal (*a.k.a*., world or generative) models that embody regularities in the organism's environment, (b) such internal models stir an organisms' interaction with the environment toward minimizing variational free energy (VFE) in sensing–acting cycles (roughly speaking, the VFE expresses prediction errors, that is, discrepancies between sensations predicted to follow actions and those actually experienced) and (c) suppression of prediction errors goes hand-in-hand with resisting entropic forces and maintaining organisms in characteristic states (of low entropy) (Friston, 2010). Our contention is that understanding engages particularly efficient mechanisms that are unique to human brains. Interested readers can find more detailed discussions of these notions in Yufik and Friston (2016) and Yufik (2019, 2021a,b). In brief:

To appreciate the distinction between understanding and learning, consider how different approaches account for superior performance in chess. The learning-centric approach attributes such performance to assimilating large stores of chess data and winning a new game with reference to the winning moves of previous games (Chase et al., 1973; Gobet and Simon, 1996). This account leaves unexplained how humans can compete with machines that have access to unlimited data and operate with processing rates billions of times faster than those seen in humans. Particularly mystifying is a quite common phenomenon of young talent defeating adult masters [e.g., a 9 year old Reshevsky played over 1,500 games of simultaneous chess in one US tour and lost <0.5% of the games (Reshevsky, 2012)]. An alternative view predicates superior performance on superior understanding. How so?

Three definitions in the literature identify significant components of the understanding capacity (with some critical exceptions, as will be explained shortly):

1. “Understanding, grasp: apprehending general relations in a multitude of particulars” (The Webster's Collegiate Dictionary).
2. “Understanding requires the grasping of explanatory and other coherence-making relationships in a large body of information. One can know many unrelated pieces of information, but understanding is achieved only when informational items are pieced together” (Kvanvig, 2003, p. 192).
3. Scientific understanding involves expressing relations in the form of equations and acquiring “some feel for the character of the solution …. if we have a way of knowing what should happen in given circumstances without actually solving the equations, then we “understand” the equation, as applied to the circumstances” (Richard Feynman, cf. de Regt, 2017, p. 102)

A simple example serves to illustrate these definitions. Consider a scene comprising just two “particulars” (dog, cat) and imagine grasping the relation between them: “dog *chasing* cat.” Note that such grasping requires (a) recognizing individual behaviors (running cat, running dog), (b) piecing these informational items together (Kvanvig, 2003, p. 192) and (c) apprehending a particular form of behavior coordination (*chase*). Grasping the relation brought about “a way of knowing what should happen in given circumstances” (Richard Feynman, c/f de Regt, 2017, p. 102) which includes prediction (e.g., if the dog runs faster than the cat it will intercept the cat; if the cat speeds up, so will the dog, etc.) and explanation (e.g., the cat is running because it's being chased by the dog). Such rough (qualitative) predictions are inherent in—and derive directly from—the relation, and can be followed by reasoning about details, in order to achieve better prediction accuracy (e.g., “solving equations” to determine the time of intercept given the distance and velocities).

Consider now increasing the number of “particulars” and complicating the scene in three ways: (a) imagine that the cat disappears behind a fence, (b) let there be an observer trying to predict what might happen and let there be a tree behind the fence, visible to the observer and (c) imagine the observer seeing no trees but entertains the possibility of their presence. In (a), the dog changes course and runs to the other side of the fence to intercept the cat. In (b) and (c), the dog's behavior does not change, but the observer realizes that the cat might climb the tree and thus leave the dog disappointed. Predators are genetically equipped with modeling mechanisms that reflect long-term statistical averages in the behavior of their prey (e.g., on the average, prey continue their movement patterns when disappearing behind objects) and allow gradual response tuning in the vicinity of such averages, based on individual experiences (learning). Such mechanisms restrict adaptive behavior to recollecting precedents—if available—or to trial-and-error, otherwise (i.e., error suppression strategies in (b) and (c) are not accessible to most creatures). By contrast, human mechanisms support the composition of unified relational structures that integrate the recollected, and current sensory elements, and simulate interdependencies among them. Understanding overcomes restrictions engendered by both genetically fixed automatisms and individual learning—and makes possible predicting and constructing adequate responses to novel conditions. The mechanism engages three key components (Yufik, 1998, 2013, 2021a,b):

1. Integration of initially unrelated elements into coherent relational models in one-step transitions (akin to phase transition in physical substrate),
2. Models are synergistic structures: they impose coordination between the constituent elements that constrain their variation,
3. Models are self-coordinating and resist fragmentation.

Some clarifications are called for here.

1. Borrowing the notion from physics, models can be viewed as *virtual systems* (Yufik, 1998) holding a superposition of possible organizations afforded by the arrangement of elements (e.g., and expert model of piece arrangements on a chessboard holds a superposition of plausible piece grouping (or functional complexes, in the sense of De Groot) (De Groot, 1965). Such superpositions collapse to one configuration yielding the steepest entropy reduction in the virtual system, giving rise to the experience of *grasp*, e.g. [(cat running somewhere), (dog running somewhere)] → (dog *chasing* cat)].
2. Collapse and compression establish coordination across the model that suppresses superfluous (redundant) variations. For example, a thought that the cat might start grooming does not cohere with the form of behavioral coordination determined by the relation, which bars such thoughts from entering the observer's mind when predicting outcomes.
3. In unified models, thinking of variations in one element effects corresponding variations in others (hence, the self-coordination). For example, envisioning the cat climbing the tree immediately implies a failure to intercept. Similarly, when considering the moves of particular pieces, unified models—held by experts—render them aware of the accompanying exposure and changing relations across the board, while fragmented models (c.f., novices) preclude such awareness (Yufik and Yufik, 2018). To intuit the difference, think of taking opponent's piece and loosing the game in a few moves (“fool's mate”) vs. sacrificing own piece and winning the game.

Crucially, compression and self-coordination in models precludes an inefficient wasting of time and energy on (considering) actions with marginal or no impact, while keeping in focus those few that decide the outcomes—the actions that “matter.” The scale of such savings can become astronomical as the number of elements increases. Studies of expert performance in complex dynamic tasks (firefighters, military commanders) have found that expert decision processes, instead of weighing alternatives, converge quickly on a single plan considered by them to be “obvious” (Klein, 2017). In a similar vein, possibilities and risks inherent in piece arrangements can be obvious to a chess prodigy, while less capable players are forced to move step-by-step through combinatorial fog. A lack of understanding turns chess positions into incoherent arrangements of pieces, each having several degrees of freedom. In contrast, expert models “squeeze out” degrees of freedom and thus provide “a way of knowing what should happen in given circumstances” (Richard Feynman, c/f de Regt, 2017, p. 102).

Summarily, understanding derives from self-organization in the brain that amplify adaptive efficiency, by supporting the construction of models representing objects, their behavior and patterns of behavioral coordination—and enabling an increase in the expressive complexity of such models, without compromising their efficient use. Stated differently, human models enable prediction and construction of apt responses to complex interplays between multiple environmental entities, by collapsing combinatorial spaces engendered by those interplays Complexity collapse (radical simplification) makes complex situations and responses to them meaningful and explainable (Yufik, 1998, 2002, 2013). Activities in neuronal masses constitutive of such models remain the subject of current and future research (Moran et al., 2013). This kind of efficiency emerges in the minimization of VFE *via* the implicit maximization of model evidence or marginal likelihood associated to the internal model. In this formulation, log model evidence can be expressed as accuracy minus complexity. This means, minimizing VFE is simply a description of the kind of sentient behavior considered above; namely providing an accurate account of exchange with the world that is as simple as possible. Understanding is the key to the right kind of complexity minimization—the right kind of collapse across degrees of freedom that capture the regularities, invariances and compositional regularities evinced by our [inter]action with the lived world. Indeed the aging brain may imbue better understanding through increased generalizability (decreased complexity, Moran et al., 2014).

We now turn to the contributions in this Research Topic. While centered on VFEM formulations, the intent for the Issue was to showcase current thinking about understanding and related problems. Accordingly, articles in the Issue address a range of opinions spanning philosophy, neuroscience, cognitive science, biology and engineering, with an excursion into biological underpinnings of cognitive pathologies. This introduction serves as an annotated table of contents, breaking the collection into several (overlapping) thematic groups.

## Philosophy of understanding

Khalifa et al. discuss the relative roles of philosophy and other disciplines (cognitive science, neuroscience, other) in advancing the science of understanding, suggesting that philosophy can offer a framework for both formulating discipline-specific accounts of understanding and then unifying such accounts under a general theory. Sloman et al. argue that inquiry into biological foundations of human intelligence should not be confined to analyzing individual brains but must consider communities of individuals.

## Understanding and consciousness

Pepperell considers whether progress in machine understanding is predicated on advances machine consciousness, leaning toward answering this question in the affirmative. Arguments encompass both general ideas and experimental findings in neuroscience, venturing into the domains of creative thinking (understanding paintings) and offering suggestions regarding the limitations of machine learning and requirements for machine understanding. Luczak and Kubo examine the relations between consciousness and adaptive efficiency. Their predictive Neuronal Adaptation hypothesis associates consciousness with prediction and ascribes prediction and error correction abilities to individual neurons—acting as basic functional units—that underwrite consciousness.

## Human-machine interaction

Parr and Pezzulo observe that applications of machine intelligence are hampered by the machine's inability to explain its decisions, and engage VEFM to argue that comprehensive explanations require the optimization of generative models at two levels: a model of the world chooses responses based on the predicted conditions in the world and a higher-level model predicts choices in the world model and uses such predictions to formulate explanations of the lower-level decisions. Schoeller et al. observe that the robustness of human-machine interaction depends on the level of trust experienced by users, and analyze trust determinants and trust-building strategies from the vantage point of VFEM. Blaha et al. point at the existence of different stages in the process of reaching understanding, and suggest natural language probes for tracing progress through the stages expected to be conserved over humans, machines and human-machine teams. Llinas and Malhotra review current research on situation control and suggest approaches, in the spirit of the VFEM, toward expanding research scope, focusing on the construction of adaptive situation models that can predict situational changes and then use prediction outcomes to minimize errors. Yufik and Malhotra. discuss distinctions and overlap in the notions of “situation awareness” and “situation understanding” and argue that attaining mutual human-machine understanding requires establishing an isomorphism between the corresponding models. More precisely, since human models represent objects, their behavior and forms of situated behavioral coordination, machine models that represent the same would be inherently explainable to users and would allow straightforward mapping of user feedback onto machine processes (hence, the mutual understanding).

## Evolutionary origins

Vicencio-Jimenez et al. discuss the thermodynamic aspects of cognitive processes and propose Energy Homeostasis Principle (EHP) complementing the VFEM principle in explaining the origins and evolution of intelligence. Intelligence develops in an open thermodynamic system (brain) in a growing hierarchy of components (neuronal groupings) that regulate their energy needs and interact with other components in the hierarchy while preserving a degree of independence. Kozma et al. rely on a vast amount of EEG data to formulate a model of neuronal processes underlying intelligence. EEG recordings demonstrate self-organization of neuronal activities, interspersed with episodic collapses in the ensuing structures. Such local phase transitions produce phase gradients that correlate with transient perceptual experiences. The mechanism of phase transition and become being gradient propagation is consistent with those envisioned in the Global Workspace Theory and may be responsible for optimizing trade-offs between demands posed by rapid adaption to novelty vs. preservation of stability. Latash discusses substantive similarities in the theories of motor control and cognitive control: both postulate predictive processes and anticipatory adjustments to actions and assume that such prediction and adjustments are carried out by self-organization processes in the control system, particularly producing task-specific synergistic groupings of control elements. These similarities may be indicative of a common synergistic mechanism participating in the entire range of control activities, “from figuring out the best next move in a chess position to activating motor units appropriate for implementing that move on the chess board” (Latash, this Research Topic).

## Cognitive architecture

Kroger and Kim investigate neuronal responses in frontopolar cortex (FPC) known to participate in the performance of complex cognitive functions, including understanding. The study seeks to determine differences in FPC involvement when subjects respond to two types of demands: acquiring and maintaining structured information vs. manipulating such information in performing cognitive tasks. Analysis of fMRI data reveals differences in FPC recruitment and activities sensitive to task organization and complexity. FPC appears to be particularly involved when responding to new and/or creating new information. Safron et al. describe a bio-inspired architecture for robotic control. Analysis of cognitive control focuses on the navigation problem involving simultaneous localization and mapping (SLAM) (i.e., build a map of the terrain concurrently with identifying one's location on the map) and hypothesizes that navigation mechanisms residing in the hippocampal/entorhinal system could be coopted by evolution in the implementation of higher cognitive functions. Construction of the world model in the SLAM architecture is governed by the VFEM principle, entailing optimization of representational units (c.f., categories) in the model.

## Machine learning

Articles in this thematic group illustrate application of machine learning methods in the type of tasks where they excel the most, i.e., classification and recognition. Cai et al. review results in the application of machine learning and feature extraction algorithms for emotion recognition, that is, classifying EEG signals and correlating such classes with emotional states of the subjects, following classifications of discrete emotional states in psychological literature. Wang and Zeng use learning in Spiking Neural Networks (SNN) to model acquisition of concepts integrating features of different sensory modalities (multisensory concept learning), under two conditions: preceding integration, inputs in each modality either become associated, or remain independent. Integration vectors produced by the SSN procedure are subsequently labeled (correlated to concepts) by psychologists.

## Neurobiological mechanisms of cognitive pathologies

Wang et al. investigate pathological conditions in the nervous system of schizophrenia patients that cause grossly maladaptive behavior (severe aggression) and admit correction only *via* medical treatment. Having established the correlation between aggression severity and inflammation accompanied by bacterial dislocation, the study suggests development of novel methods for containing aggression, which focus on suppressing inflammation.

## Summary and conclusions

To summarize, the articles in this collection present partially overlapping as well as strongly diverging opinions on issues dealing with intelligence and adaptive efficiency in a wide range of settings, from social groups to human-machine teams and down to individuals demonstrating performance varying from superior to pathological. The VFEM principle applies at all levels to some degree; from adjusting social policies, correcting individual behavior, and treating pathologies. Understanding is an adaptive strategy within the VFEM scope, expressing integrative operation of two core principles, as follows.

Models represent regularities in the record of sensory inflows and an organism's responses, and vary in scope: from representing contiguous elements in short segments in the record to representing non-contiguous element groupings separated by indefinitely large segments (Yufik, 1998, 2018; Yufik and Sheridan, 2002). Regularities constitute compressible components in the record, with the degree of compression dependent on the types of pressure that drive adaptation. In particular, environmental pressure demands minimization of prediction errors (i.e., VFE) consequent on the organism's decisions, while thermodynamic pressure demands maintaining life-compatible ratios of energy intakes vs. energy expenditures in the brain producing those decisions. The adaptation-by-learning strategy (recall and compare) subsists on low degrees of compression, limiting adaptation scope to low-complexity contingencies in the organism's vicinity (think of predators chasing preys). By contrast, a uniquely human genetic pressure (i.e., curiosity and the desire to understand) requires unlimited expansions of expressivity over spatial, temporal and complexity dimensions—thus creating an incessant demand for compression and the minimization of complexity (think of formulating theories and aha moments when the simplicity of the solution reveals itself).

Biophysics imposes hard constraints on brain development, limiting the size of the neuronal pool and the ratio of energy supply and expenditure compatible with sustaining life. Complexity and thermodynamic (and metabolic) constraints are intimately linked. For example, the Jarzynski equality tells us immediately, that the more we change our mind—in terms of erasing information—the more energy we consume. Technically, this enables one to associate the complexity of our world models with the metabolic cost of maintaining them in open exchange with the environment. Grasp (abrupt unification of disparate neuronal processes in coherent and self-coordinating structures) aptly responds to all three forms of pressure under complexity and thermodynamic constraints, i.e., grasp mechanisms allow unlimited expansion in the scope of regularities captured in world models, while yielding adequate prediction accuracy at sustainable energy costs. Grasp extracts the essence (the gist) of a situation, enabling predictions at costs that are infinitesimally small in comparison with those the system would be facing without grasp. To fully appreciate the scale of savings, think of 15 moves look-ahead reported by world class masters (Kasparov, 2007). Shannon's (1950) formula puts the number of possible games after 15 half-moves at approximately 2 × 10^21^. Making an assumption that a player can evaluate one such possibility per second and can keep this rate up for 30 mins obtains about 2 × 10^3^ evaluations, indicating reduction in the amount of processing on the scale 10^18^:1. Figuratively, grasp confines costly evaluations (reasoning about moves) to the gist of the position held within a hair thin path in a combinatorial ocean that is million times wider than the Pacific. Some articles in this Issue resonate with the above ideas, while some others offer interesting alternatives.

In conclusion, we offer some observations and suggestions for future research in biological and machine intelligence. The history of the latter can be divided into four periods: pebbles, abacus, calculators and computers. Gadgets of the former three types hold only data, while algorithms for manipulating data remain in the mind of the user. The computer revolution was propelled by the realization (John von Neumann) that algorithms can be held alongside data in the same medium. This revolution allowed the delegation of learning to machines, with the temptation to reduce all of higher cognition to algorithmic data manipulation (machine learning). As a result, progress in machine intelligence has relied primarily on advances in the efficiency of data manipulation, which is, in a way orthogonal to that exploited by evolution (human neurons are not faster, smaller or more energy efficient than other species, though there are more of them). The tremendous value produced by machine learning does not change the fact that, in principle, learning machines operate in a context invariant fashion—in familiar conditions—and can only deceive users into ascribing understanding to them while, in fact, having none.

Evolution has explored the adaptation-by-learning route in millions of species and during billions of years since the emergence of life on earth, and ran into a dead end in higher animals. Understanding is a product of a recent evolutionary discovery [which, conceivably, coopted some existing mechanisms (Yufik, 2018, 2021a) that, in about 100,000 years, advanced human civilization from foraging and hunting to launching missiles and sending telescopes to the outer space]. The core mental act of ‘merging pieces together' is non-verbalizable but could have given birth to language (Berwick and Chomsky, 2017). The adaptive value of a non-algorithmic “grasp” derives precisely from its ability to overcome inertia and dissolve templates acquired in the course of learning. It is not unreasonable to assume that imparting a modicum of understanding capacity to machines could bring about benefits on a par with or greater than those delivered by the computer revolution.

Technically speaking, the transition from machine learning to machine understanding shifts the research emphasis from representing recognition *via* vector mapping (as in neural nets) to representing relations *via* coordinated vector movement (think of the domino row and associate direction vector with each piece—considering that rotating one vector in the first piece brings about similar rotations in others). Challenges posed by deviating from the von Neumann–Turing architecture and/or designing computable approximations of the ways understanding operates might be stupendous but not insurmountable (Siegelmann, 1999; Yufik, 2002; Traversa and Di Ventra, 2017; Di Ventra and Traversa, 2018; Hylton, 2022). VFEM does not stipulate methods for implementing machine intelligence but constrains the conceptual or computational space for formulating them and establishes a tractable performance metric. Arguably, the problem of machine consciousness is subordinate to that of machine understanding: if understanding is a lens, consciousness acts as an eyelid: one can see when the lid is up and not when it is down (with degrees of clarity depending on the degree of squinting).

A recent book on expert decision making was entitled “Sources of Power” (Klein, 2017), whose title coheres with one of the key insights in a philosophical classic:

“Quite generally, the familiar, just because it is familiar, is not cognitively understood. The commonest way in which we deceive either ourselves or others about understanding is by assuming something is familiar and accepting it on that account; with all its pros and cons, such knowing never gets anywhere, and it knows not why.

… The analysis of an *idea*, as it used to be carried out was, in fact, nothing else than ridding it of the form in which it had become familiar. …The activity of dissolution is the power and work of the *Understanding*, the most astonishing and mightiest of powers, or rather the absolute power” [Hegel, [1977 (1807)], p. 18].

Harnessing this power can be decisive in securing competitive edge in commerce and defense.

## Author contributions

All authors listed have made a substantial, direct, and intellectual contribution to the work and approved it for publication.

## Author's disclaimer

The views expressed in this article are solely those of the authors and do not necessarily represent those of the United States Air Force.

## Conflict of interest

Author YY was employed by Virtual Structures Research Inc. The remaining authors declare that the research was conducted in the absence of any commercial or financial relationships that could be construed as a potential conflict of interest.

## Publisher's note

All claims expressed in this article are solely those of the authors and do not necessarily represent those of their affiliated organizations, or those of the publisher, the editors and the reviewers. Any product that may be evaluated in this article, or claim that may be made by its manufacturer, is not guaranteed or endorsed by the publisher.
